# Performance Analysis of a Travelling-Wave Ultrasonic Motor under Impact Load

**DOI:** 10.3390/mi11070689

**Published:** 2020-07-16

**Authors:** Jiahan Huang, Dong Sun

**Affiliations:** 1 School of Mechatronics Engineering, Foshan University, Foshan 528225, China; jiahan1989@126.com; 2 School of Mechanical Engineering, Nanjing University of Science and Technology, Nanjing 210094, China

**Keywords:** shock environment, ultrasonic motor, dynamic response, mechanical characteristic

## Abstract

With the increased application of ultrasonic motors, it is necessary to put forward higher demand for the adaptability to environment. Impact, as a type of extreme environment, is widespread in weapon systems, machinery and aerospace. However, there are few reports about the influence of impact on an ultrasonic motor. This article aimed to study the reasons for the performance degradation and failure mechanism of an ultrasonic motor in a shock environment. First, a finite element model is established to observe the dynamic response of ultrasonic motor in a shock environment. Meanwhile, the reasons of the performance degradation in the motor are discussed. An impact experiment is carried out to test the influence of impact on an ultrasonic motor, including the influence on the mechanical characteristic of an ultrasonic motor and the vibration characteristic of a stator. In addition, the protection effect of rubber on an ultrasonic motor in a shock environment is verified via an experimental method. This article reveals the failure mechanism of ultrasonic motors in a shock environment and provides a basis for the improvement of the anti-impact property of ultrasonic motors.

## 1. Introduction

Over the past few decades, ultrasonic motors (USMs) based on the piezoelectric effect have been developed well. There are many industrial applications, including micro-machine, information technology, surgery devices, ecological/energy areas, absolute gravimeter and nano-positioning stages [1,2,3,4,5]. There are numerous merits of USMs over electromagnetic motors, including low speed with high torque, a quick response, a wide velocity range and a high power/weight ratio [2,3,4,6]. The unique properties of USMs make it have a bright application prospect in extreme environments, such as space exploration [7,8]. A lot of research reports, mainly focusing on the application of USMs with ambient temperature changes and under vacuum, have been released [9,10,11,12,13,14,15,16].

In order to expand the application range of ultrasonic motors, some researchers start to turn their sights onto weapon systems, such as smart wings in airplanes [4], driving and anti-stealth technology [17] and a smart fuse safety system [18,19]. Impact, widespread in ammunition system, is an unavoidable destructive factor that needs to be considered. Many scholars have carried out a lot of research about various devices in a shock environment, such as the reliability of MEMS devices [20,21,22] and data recorders used in ammunition [23,24]. However, there are only a few research reports about the characteristic of an ultrasonic motor in a shock environment. Ren et al. tested the mechanical characteristic of ultrasonic motor after impact test, but there is a lack of analysis about the failure mechanism [25]. Our previous research established a model about the dynamic response of an ultrasonic motor in a shock environment based on the rigid plasticity model [26]. The model reflects the dynamic process to a certain extent, but more accurate results are needed to analyze the characteristic of an ultrasonic motor in a shock environment.

This article aimed to study the reasons for the performance degradation and failure mechanism of an ultrasonic motor in a shock environment. A finite element model, which takes into account material nonlinearity, is established. The dynamic response of an ultrasonic motor in a shock environment can be observed through transient solution. Meanwhile, the relationship between structural plastic deformation and preload is obtained to explain the performance degradation. The mechanical characteristic of an ultrasonic motor after impact is tested. Moreover, the compensation gaskets are applied to recover the performance of an ultrasonic motor. Meanwhile, the distortion of the vibration characteristic of a stator after impact is discussed. Finally, the protection effect of rubber for an ultrasonic motor in a shock environment is verified by experiment.

## 2. Structure of an Ultrasonic Motor

**Figure 1:**
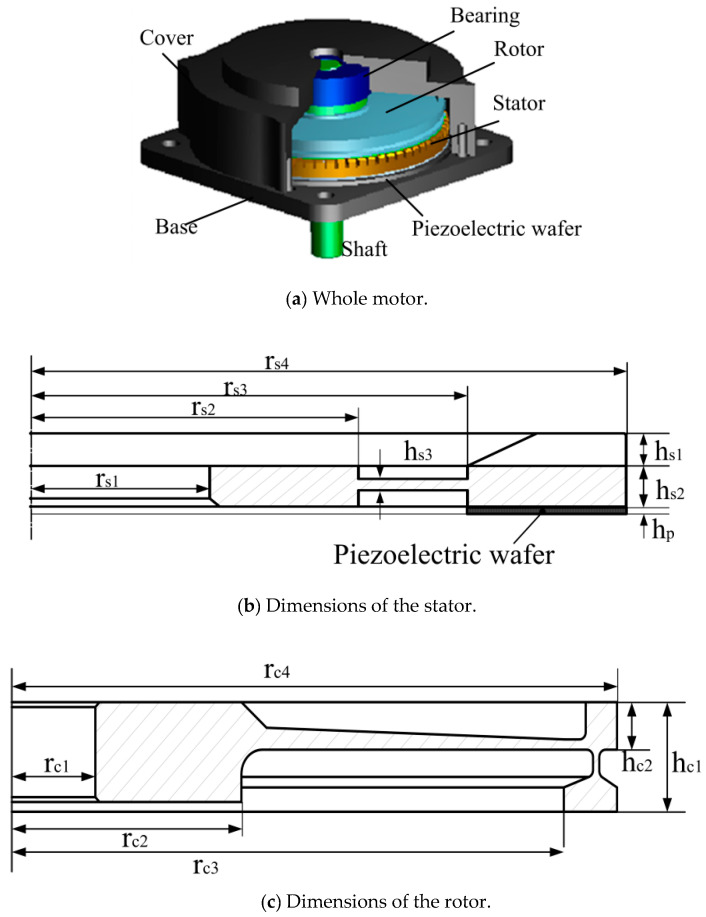
Typical structure of a travelling wave rotary ultrasonic motor.

The typical structure of a travelling-wave ultrasonic motor is shown in Figure 1a [27]. The ring-shaped stator with many teeth to enlarge the vibration amplitude is fixed on the base. The piezoelectric wafer is affixed to the bottom surface of the stator. A rotor is on the top of stator. The cover is fixed on the base. The bearing is used to improve the work efficiency by reducing friction while operating. There is preload between the stator and rotor by applying a gasket between rotor and bearing. By applying electric signal with a frequency close to the resonance frequency of the stator, there is a continuous elliptical motion in the top of the stator. This motion is converted to the movement of the rotor through the friction between the stator and rotor [28].

The operating mechanism of an ultrasonic motor consists of two energy conversion processes: One is the conversion of electrical energy into mechanical energy of high-frequency and micro-amplitude vibration in stator through the inverse piezoelectric effect of piezoelectric materials. The other converts the micro-vibration of the stator into the macroscopic motion of the rotor through the friction between them. After impact, the damage to the piezoelectric wafer or stator, including the fracture of the piezoelectric wafer and the plastic deformation of the stator, may lead to the incomplete vibration mode of the stator, so that the input power cannot be converted into high-frequency and micro-amplitude vibration of the stator efficiently. On the other hand, the plastic deformation of the rotor or stator will lead to a decrease in preload. The analysis of the two convention processes in a shock environment can be considered as the analysis of deformation of the structure and anti-impact property of the stator and piezoelectric wafer. 

### Model Establishment

**Figure 2:**
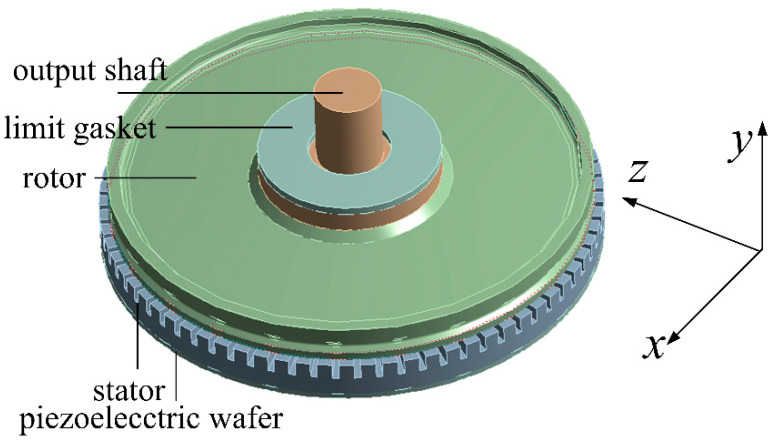
Finite element model.

As a complex electromechanical coupling system, transient analysis is necessary to observe the dynamic response of an ultrasonic motor in a shock environment. The model is established in Workbench 14.0. The key components that cannot be ignored are the stator, piezoelectric wafer and rotor. Instead, the base and cover can be ignored because their strength is large enough to withstand impact damage. The cover will limit the upward movement of the rotor in a shock environment. In order to show this phenomenon accurately, a limit gasket is set on the top of the rotor to limit the upward movement of the rotor, as shown in Figure 2. To reduce the amount of calculation and computational complexity, the mass of the shaft in the simulation was equivalent to a combination of a bearing and output shaft.

Ultrasonic motor TRUM60, which means the diameter of the stator is 60 mm, is selected. The key dimensions of the stator (Figure 1b) and rotor (Figure 1c) are listed in Table 1. Moreover, it is significant to know the weight of the shaft, because it might increase the risk of rotor damage in a shock environment. 

**Figure 3:**
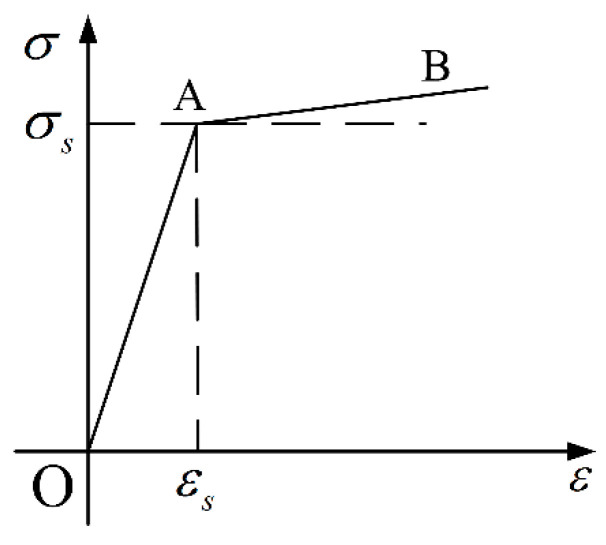
Bilinear isotropic hardening model.

There are several kinds of materials, including phosphor bronze for the stator, aluminum for the rotor, PZT-4 for the piezoelectric wafer, and structure steel for the shaft. Structural deformation is what we are concerned about during the analysis process, especially in the stator and rotor. The bilinear isotropic hardening model, as a classic elasto-plastic mechanics model, is widely used in engineering research. The mechanical property of the material model is shown in Figure 3. Assume that the material models of the rotor and stator are bilinear isotropic hardening materials. Assume structural steel to be an isotropic material, because the output shaft is strong enough to withstand impact damage. A piezoelectric wafer is a kind of anisotropic material. Compared to the whole USM, the volume and mass of a piezoelectric wafer can be neglected, so it has an extremely low impact on the dynamic characteristics of a USM in shock environment. Therefore, to reduce the amount of calculation and computational complexity, the piezoelectric ceramic is simplified into an isotropic material. Adhesive and friction material are not taken into consideration here. The key material parameters are shown in Table 2.

Besides, there are some contact and boundary conditions that need to be set. The grid of the contact area is made to be high quality. The output shaft and rotor are bonded together to replace the threaded connection. The displacement of the inner boundary of the stator is constrained to zero at all degrees of freedom. Assume that the structure has experienced a half sine shock acceleration, which can be expressed as:

(1) $$a(t)=\{\begin{array}{c} a_{0}\sin(\frac{\pi }{\tau }t),0\leq t\leq \tau \\ 0,t>\tau \end{array}$$

where *a*_0_ represents the amplitude of shock acceleration and *τ* represents the pulse width of shock acceleration.

## 3. Dynamic Responses of an Ultrasonic Motor

### 3.1. Modal Analysis 

**Figure 4:**
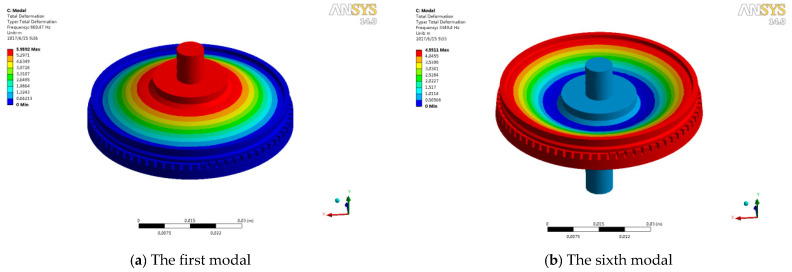
First and sixth natural frequency of an ultrasonic motor.

The finite element modal analysis of the whole structure is carried out. The results are shown in Figure 4. Because the direction of impact follows the Y axis, the mode shapes in the Y direction are selected. The frequency of first modal (Figure 4a) and sixth modal (Figure 4b) is 860.47 Hz and 3349.4 Hz, respectively. The output shaft cannot remain still in a shock environment considering the structure of an ultrasonic motor. The first modal with a frequency of 860.47 HZ is more easily stimulated in a shock environment.

### 3.2. Dynamic Response of an Ultrasonic Motor

**Figure 5:**
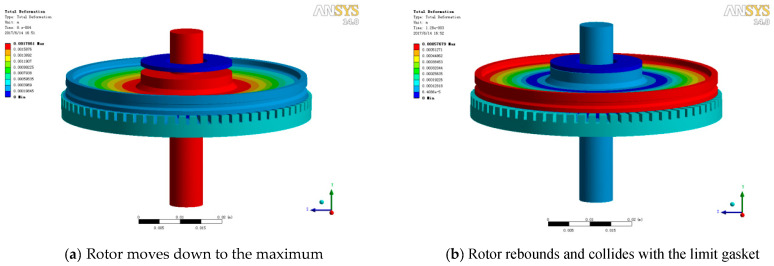

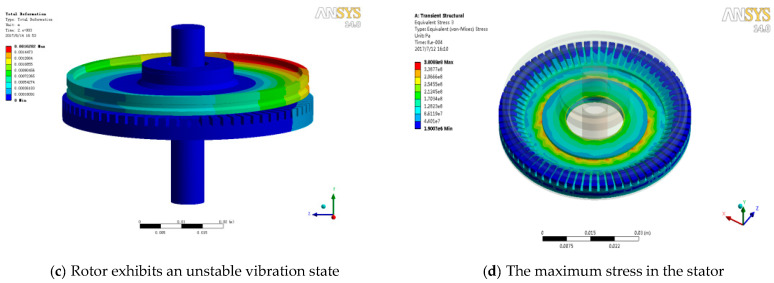
Dynamic response of an ultrasonic motor and stress distribution in a stator.

**Figure 6:**
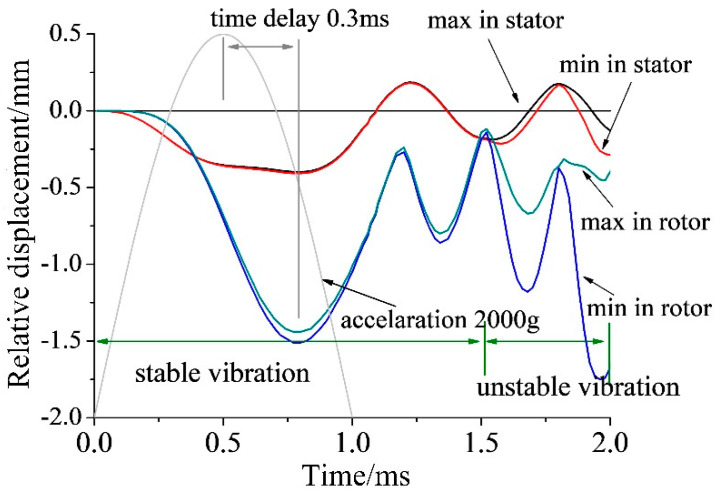
Simulated displacement-–time curves of a rotor and a stator.

By applying a half sine acceleration pulse with an amplitude of 2000 g and a pulse width of 1.0 ms, transient simulation has been done. The dynamic responses at different times are listed in Figure 5. The shaft moves down to the maximum, as shown in Figure 5a. Due to the elastic effect of the structure, the rotor rebounds and then collides with the limit gasket, as shown in Figure 5b. Then, the whole structure turns into a complex transient vibration state. Multiple collisions between rotor, stator and limit gasket result in structural instability, especially in the rotor. The rotor exhibits an unstable vibration state, as shown in Figure 5c. The maximum stress in the stator is 380 MPa at a time of 0.8 ms during the whole process, as shown in Figure 5d, which is smaller than the yield stress. It means that there is no plastic deformation in the stator. Actually, the anti-impact property of the stator is far greater than the rotor because of the structure difference. Figure 6 shows the displacement-time curves of the rotor and stator. Curves “max in rotor” and “min in rotor” refer to the relative displacement between the upper surface of the inner ring and the bottom surface of the outer ring in the rotor. Obviously, there is irreversible plastic deformation in the rotor. Because the inner boundary of the stator is constrained, the curves “max in stator” and “min in stator” refer to the displacement of the outer edge of the bottom surface in the stator. The dynamic response of the structure can be divided into two parts: Period 1, the outer ring of rotor maintains a stable ring, and the center part moves up and down with the shaft; Period 2, the outer ring of the rotor is no longer stable, which leads to the result that “max in rotor” and “min in rotor” gradually separate. The peak value of the displacement has a time delay of 0.3 ms with the peak value of the impact, as shown Figure 6.

### 3.3. Dynamic Response of an Ultrasonic Motor in a Shock Environment with a Different Amplitude and Pulse Width 

**Figure 7:**
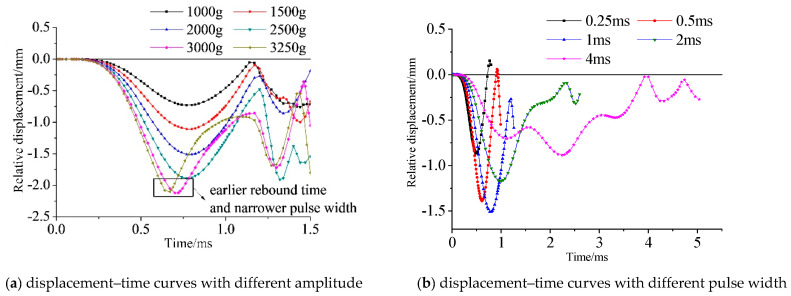
Simulated displacement–time curves of a rotor under impact with a different amplitude and duration.

Dynamic responses do not only depend on the amplitude of shock acceleration but also the pulse width. Compared with the stator, the rotor with lower strength is easier to distort. Hence, the dynamic response of the rotor is what concerns us. Figure 7a shows the results of the maximum relative displacement–time curves of the rotor under an impact of 1000 g, 1500 g, 2000 g, 2500 g, 3000 g, and 3250 g with a pulse width of 1 ms. Apparently, the deformation increases with the increase in the amplitude. While the impact exceeds 3000 g, the center part of the rotor collides with the stator and bounces back. It will result in an earlier rebound time and a shorter pulse width. Figure 7b shows the results of displacement–time curves of the rotor under an impact of 2000 g with a pulse width of 0.25, 0.5, 1, 2, and 4 ms. Impact with a pulse width of 0.5 ms and 1 ms will cause maximum displacement in the rotor. Pulse widths of 0.5 and 1 ms correspond to the frequencies 1000 and 500 Hz, which are closest to the first modal frequency of 860.47 Hz among all the pulse widths listed above. This phenomenon is consistent with the results of the modal analysis.

### 3.4. Relationship between Plastic Deformation in Rotor and Preload

**Figure 8:**
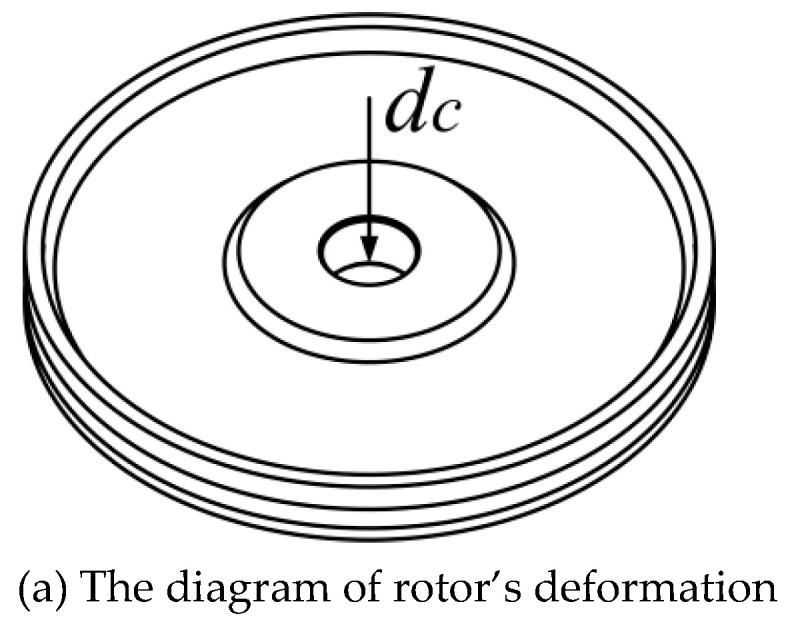

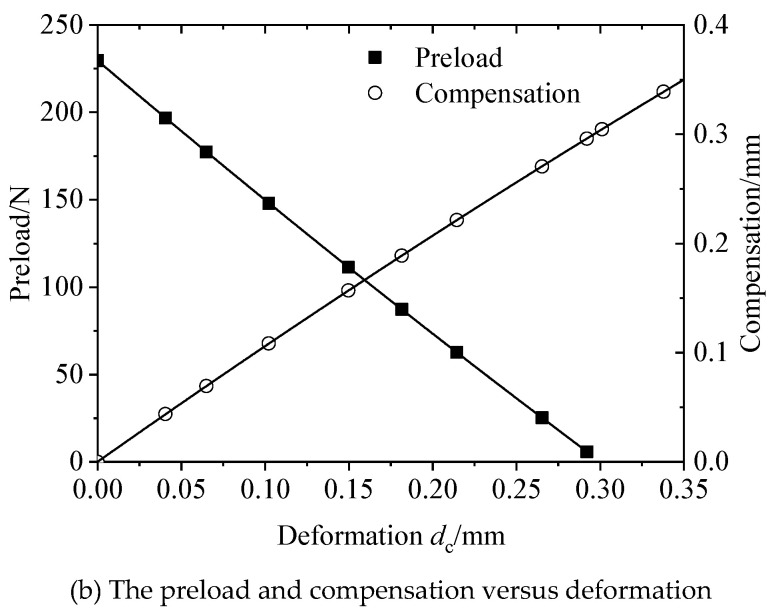
Relationship between deformation again preload and compensation.

The micro-vibration of the stator can be transferred into the macroscopic motion of the rotor through the friction between them. The preload between stator and rotor is produced through a gasket between the rotor and bearing. Based on finite element calculation, the preload is 229.71N while the thickness of gasket is 0.3 mm. Many theoretical and experimental results have proved that the preload is significant to the performance of motor [29,30]. Obviously, the plastic deformation of the rotor will lead to a decrease in preload. Assume that sinkage in the center of rotor is *d*_c_ after impact, as shown in Figure 8a. By applying a static acceleration that will result in plastic deformation in the rotor, the relationship between deformation *d*_c_ and preload under the premise of a gasket with a constant thickness of 0.3 mm is obtained, as shown in Figure 8b. Obviously, there is a relationship between *d*_c_ and the preload. The polynomial fitted formula is:

(2) $$y_{\mathrm{preload}}=229.60-813.92d_{c}+163.93d_{c}^{2}$$

where *y*_preload_ is the preload, *d*_c_ is the deformation of the rotor. It is necessary to recover the preload via adding the thickness of the gasket to restore the performance of the motor. The thickness that ensures the preload recover to the initial value is called “compensation”, and the relationship between *d*_c_ and compensation is calculated by the finite element method, as shown in Figure 8b. The polynomial fitting formula is:

(3) $$y_{\mathrm{compensation}}=1.76\times 10^{-4}+1.08d_{c}-0.234d_{c}^{2}$$

where *y*_compensation_ is the thickness of the compensation gasket and *d*_c_ is the deformation of the rotor.

## 4. Experiments 

**Figure 9:**
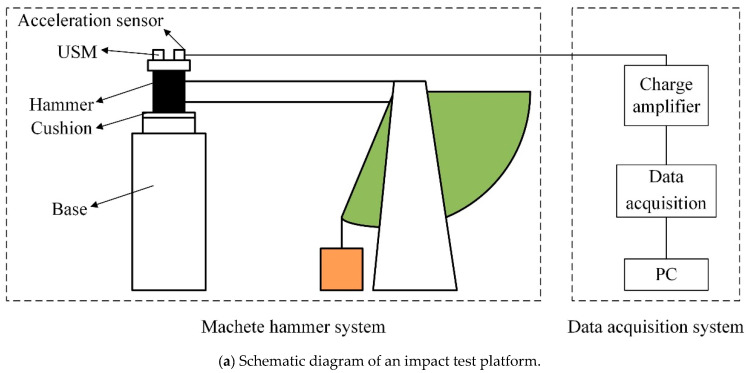

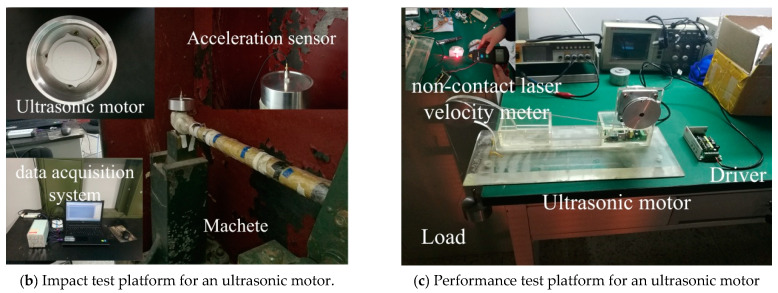
Experiment test platform.

Three substantially identical ultrasonic motors (TRUM60) are chosen to test the performance change after impact test. Ultrasonic motors are fixed on the fixture. The fixture is fixed on a Machete hammer to carry out the impact test. The half-sine acceleration pulse induced by the Machete hammer is obtained by free falling on the cushion, and the half-sine accelerations with a different amplitude can be obtained by adjusting the height of the hammer and the thickness of the cushion. The whole USM is fixed on the hammer through the clamp device, and the shock acceleration is applied to the whole USM. The schematic diagram of the shock experiment is shown in Figure 9a. A data acquisition system is adopted to capture the acceleration data. The impact test platform is shown in Figure 9b. A mechanical characteristic test platform is set up to test the performance of the ultrasonic motor after impact, as shown in Figure 9c. The speed of motors is measured under different loads utilizing a non-contact laser velocity meter.

### 4.1. Mechanical Characteristic Test and Result Analysis

**Figure 10:**
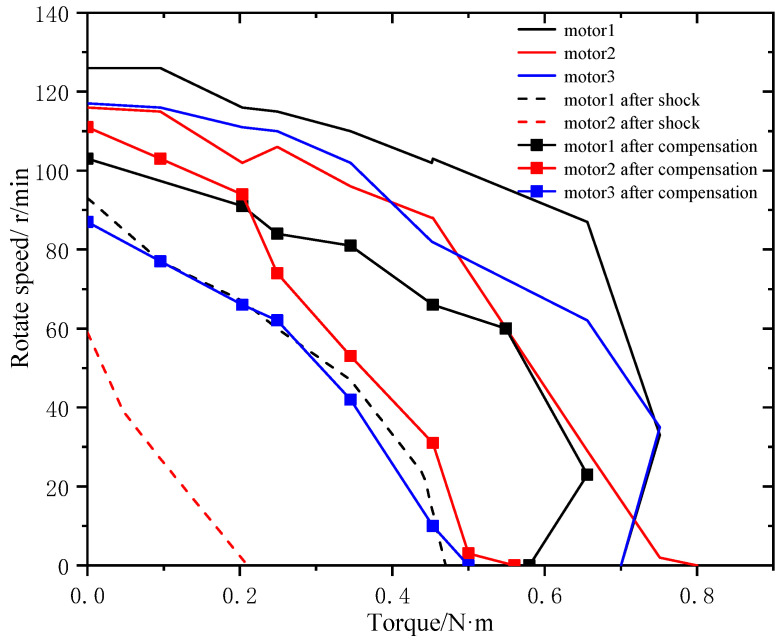
Performance of motors before and after impact and compensation.

Different acceleration amplitudes of 1630, 1865 and 2320g are applied to three motors numbering 1 to 3, respectively. The original mechanical characteristic of the motors is shown in Figure 10. After impact, ultrasonic motor 1 can work normally, but the output performance degrades. Performance degradation in motor 2 is more apparent and there is noise “sha-sha” while operating. Motor 3 cannot work while powered. The output shaft can rotate freely, which means that the preload disappears and the motor has no self-locking ability. The mechanical characteristic of motors after impact is shown in Figure 10. 

Obviously, the central part of the rotor is sunken, and the depth of sinking is measured and listed in Table 3. The theoretical preload after impact based on Equation (2) is listed in Table 3. The compensation is carried out to restore preload. The theoretical compensation thickness is based on Equation (3), and the actual thickness of the compensation gaskets is listed in Table 3. The actual compensation thicknesses are obtained through debugging when ultrasonic motors are at optimal performance levels. There is a difference between theoretical and actual compensation. Different thickness gaskets are used in debugging. The voids between gaskets lead to the increase in total thickness, which means the decrease in actual thickness.

Moreover, the performance of motors after compensation is tested, as shown in Figure 10. It is obvious that the mechanical characteristic will be restored with the application of the compensation gasket. However, the performance will never be restored to original state. It will always degrade irreversibly. The reasons behind this need more analysis.

### 4.2. Anti-Impact Property of Stator

**Figure 11:**
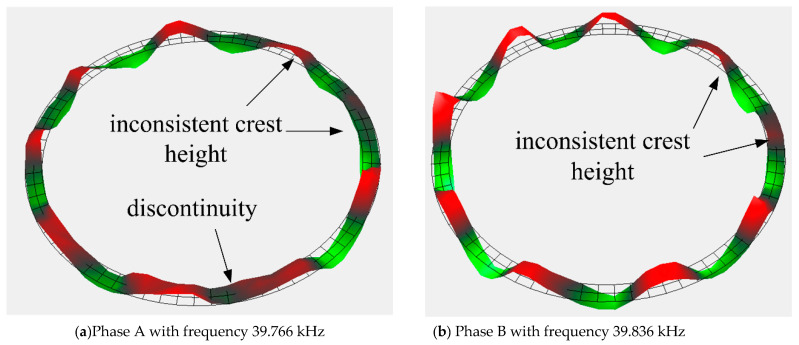
Vibration characteristic of stator 1.

**Figure 12:**
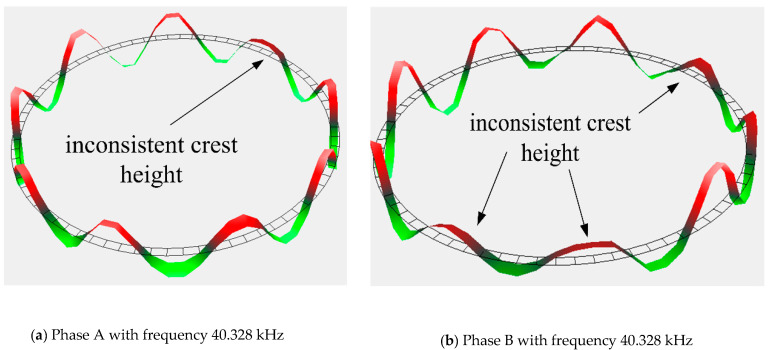
Vibration characteristic of stator 2.

In order to test the anti-impact property of stators, motors 1 and 2 are subjected to a higher impact load of 9718.3 and 3582.6 g, respectively. Obviously, the two motors cannot operate normally. There is no obvious damage in the stators. The vibration characteristic of the stators after impact is measured by a laser Doppler vibrometer system (PSV-300F-B). The vibration shapes of stator 1 and 2 are relatively complete, as shown in Figure 11 and Figure 12. However, the vibration shape of stator 2 is far better than that of stator 1. The crest heights in these two stators are both inconsistent, but the inconsistence is more apparent in stator 1. Besides that, there is a wave discontinuity in stator 1. The inconsistent crests show that the stator presents a state of twisted vibration, which means that the stator is no longer a stable ring. This will lead to inhomogeneous contact between the stator and rotor; thus, the output torque and speed of the USM are severely weakened. The small distortions in the vibration characteristic of stators are part of the reasons behind the performance degradation after the compensation described in previous section.

**Figure 13:**
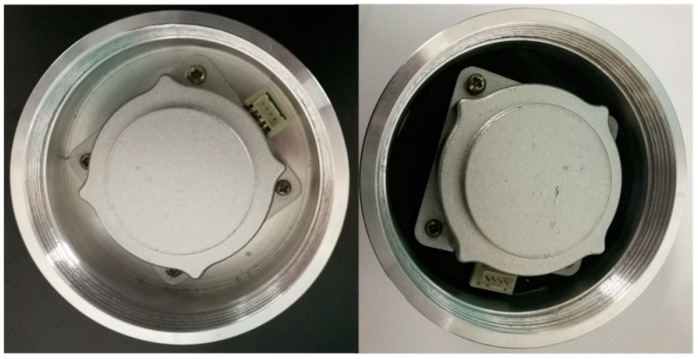
Ultrasonic motor with (right) and without (left) the protection of rubber.

**Figure 14:**
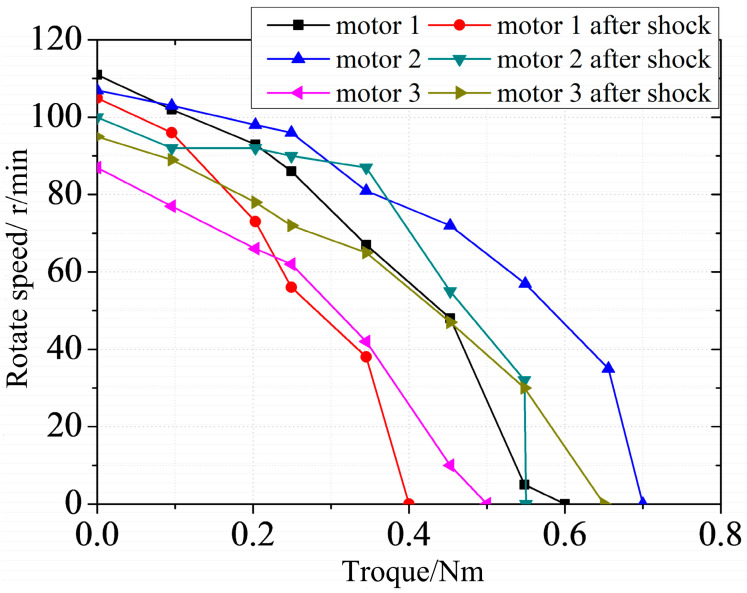
Performance of motors with the protection of rubber.

In order to ensure the performance of ultrasonic motor in shock environment, the protection device is necessary. Rubber, which can effectively reduce the impact amplitude and increase the impact pulse width, is widely used in vibration and impact buffering devices because of its excellent property and its cheap price. A shock experiment is carried out with the protection of rubber. The impact loads are 3090, 3035, and 2980 g. The ultrasonic motors used in the experiment are the three motors that have been adjusted after the previous experiment. The rubber used during the experiment is shown in Figure 13. The mechanical characteristic of the ultrasonic motor is tested after impact with the protection of rubber. The experiment results show that the performance of the ultrasonic motor drops slightly compared with that has no protection method, as shown in Figure 14. Obviously, rubber can protect the motors in a shock environment to some extent.

## 5. Conclusion

In this article, a typical structure of a travelling-wave ultrasonic motor is proposed and the influence of shock acceleration on an ultrasonic motor is investigated. A theoretical model is established to observe the dynamic response of an ultrasonic motor in a shock environment based on the finite element method. Meanwhile, a relationship between plastic deformation in the rotor and preload is obtained. After that, an impact experiment is carried out to test the influence of a shock environment on an ultrasonic motor, including the influence on the mechanical characteristic of motors and the vibration characteristic of stators. Moreover, the compensation gasket is applied to recover the performance of motors. Finally, the protection effect of rubber on an ultrasonic motor in a shock environment is verified by experiments. The meaningful results are summarized in the following.

The deformation of structure does not only depend on the amplitude of shock acceleration but also the pulse width. The distortion of the rotor is smaller while the shock frequency is far away from the resonant frequency. This means that the influence of shock acceleration on an ultrasonic motor can be reduced by changing the impact pulse width besides reducing impact amplitude. 

The preload decreases while the deformation of the rotor increases. It will result in the performance degradation of the motor. The preload can be recovered by adding an extra gasket. The relationships between the deformation of the rotor and preload as well as compensation thickness are obtained, respectively. The experiment results also show that the mechanical characteristic can be recovered by applying an extra gasket.

A shock environment will lead to the small distortion of the vibration characteristic of a stator, including the inconsistent crest heights and wave discontinuity. This is part of the reason that leads to irreversible damage to the ultrasonic motors. As a typical cushioning material, rubber can protect an ultrasonic motor in a shock environment efficiently. A protection device is necessary when an ultrasonic motor is applied in a shock environment.

## Figures and Tables

**Table 1:** Key parameters of a travelling-wave ultrasonic motor (unit: mm).

Parameter	Value	Parameter	Value
r_s1_	9	r_s2_	16.5
r_s3_	22	r_s4_	30
h_s1_	2	h_s2_	2.5
h_p_	0.5	r_c1_	4
r_c2_	11	r_c3_	27.5
r_c4_	29	h_c1_	4.5
h_c2_	2	weight of shaft	23g

**Table 2:** Material parameters.

Material	Density (kg/m^3^)	Young’s Modules (Pa)	Poisson’s Ratio	Yield Strength (Pa)	Tangent Modulus (Pa)
aluminum	2770	7.1 × 10^10^	0.33	2.8 × 10^8^	5 × 10^8^
phosphor bronze	8760	1.12 × 10^11^	0.33	4.4 × 10^8^	1.15 × 10^9^
Piezoelectric wafer	7650	3.5 × 10^10^	0.31	*	*
Structural steel	7850	2 × 10^11^	0.3	*	*

**Table 3:** Deformation dc of a rotor and compensation thickness.

Number	Deformation d_c_ (mm)	Theoretical Preload after Impact(N)	Theoretical Compensation (mm)	Actual Compensation (mm)
1	0.1	149.8	0.106	0.1
2	0.2	73.4	0.207	0.15
3	0.55	0	0.523	0.45
